# Reduction of spondylolisthesis and restoration of lumbar lordosis after anterior lumbar interbody fusion (ALIF)

**DOI:** 10.1186/s12893-023-01966-z

**Published:** 2023-03-27

**Authors:** Stefan Aspalter, Harald Stefanits, Christoph Johannes Maier, Christian Radl, Helga Wagner, Philipp Hermann, Martin Aichholzer, Nico Stroh, Andreas Gruber, Wolfgang Senker

**Affiliations:** 1 Department of Neurosurgery, Kepler University Hospital Linz, Johannes Kepler University, Linz, Austria; 2 Center for Clinical Studies (CCS Linz), Johannes Kepler University, Linz, Austria; 3 Department of Medical Statistics and Biometry, Institute of Applied Statistics, Johannes Kepler University, Linz, Austria; 4 Department of Neurosurgery, Neuromed Campus, Kepler University Hospital, Wagner-Jauregg Weg 15, Linz, 4020 Austria

**Keywords:** Spondylolisthesis, Lordosis, Spinal fusion, Lumbar vertebrae, Vascular system injuries

## Abstract

**Background:**

Anterior lumbar interbody fusion (ALIF) is a well-established surgical treatment option for various diseases of the lumbar spine, including spondylolisthesis. This study aimed to evaluate the postoperative correction of spondylolisthesis and restoration of lumbar and segmental lordosis after ALIF.

**Methods:**

Patients with spondylolisthesis who underwent ALIF between 2013 and 2019 were retrospectively assessed. We assessed the following parameters pre-and postoperatively (6-months follow-up): Visual Analogue Scale (VAS) for pain, Oswestry Disability Index (ODI), lumbar lordosis (LL), segmental lordosis (SL), L4/S1 lordosis, and degree of spondylolisthesis.

**Results:**

96 patients were included. In 84 cases (87.50%), additional dorsal instrumentation was performed. The most frequent diagnosis was isthmic spondylolisthesis (73.96%). VAS was reduced postoperatively, from 70 to 40, as was ODI (50% to 32%). LL increased from 59.15° to 64.45°, as did SL (18.95° to 28.55°) and L4/S1 lordosis (37.90° to 44.00°). Preoperative spondylolisthesis was 8.90 mm and was reduced to 6.05 mm postoperatively. Relative spondylolisthesis was 21.63% preoperatively and 13.71% postoperatively. All clinical and radiological improvements were significant (all p < 0.001). No significant difference considering the lordosis values nor spondylolisthesis was found between patients who underwent ALIF surgery without dorsal instrumentation and patients who received additional dorsal instrumentation. Venous laceration was the most frequent complication (10.42%).

**Conclusions:**

With ALIF, good clinical results and safe and effective reduction of spondylolisthesis and restoration of lordosis can be achieved. Additional dorsal instrumentation does not significantly affect postoperative lordosis or spondylolisthesis. Individual vascular anatomy must be reviewed preoperatively before considering ALIF.

## Introduction

Anterior lumbar interbody fusion (ALIF) is a well-established surgical treatment option for various lumbar spine pathologies. Advantages compared to posterior approaches to the spine are reduced trauma to the paraspinal muscles and avoidance of entrance into the spinal canal and retraction of nerve roots, and consequently, no postoperative epidural fibrosis and scarring. Also, good visualization of the anterior column with easy access for complete discectomy is possible [1].

ALIF is an effective treatment for spondylolisthesis as it stabilizes the spinal column and decompresses nerve roots indirectly through the restoration of disc height [1]. ALIF can be used in cases of isthmic spondylolisthesis as well as degenerative spondylolisthesis. While some authors routinely use ALIF alone for spondylolisthesis cases [2, 3], others combine it with dorsal instrumentation for these patient groups [4]. Dorsal instrumentation also provides better long-term fusion rates [5].

Most studies investigating ALIF and the extent of correction of spondylolisthesis and lordosis have a low case number. Also, studies comparing ALIF alone and ALIF with additional dorsal instrumentation are rare. Therefore, this retrospective, monocentric study aimed, firstly, to evaluate the reduction of spondylolisthesis and restoration of lordosis in a real-life selection of patients who underwent single-level ALIF surgery, either alone or combined with dorsal instrumentation, and secondly, to describe differences between the standalone and with dorsal instrumentation groups.

## Methods and materials

The research related to human use has been complied with all the relevant national regulations, institutional policies and in accordance the tenets of the Helsinki Declaration, and has been approved by the authors’ institutional review board or equivalent committee. We obtained approval for the study from the ethics committee of the Federal State of Upper Austria (1273/2019). We carried out a retrospective analysis of patients who underwent ALIF at our department between 1.1.2013 and 10.12.2019. Only patients who showed a measurable amount of spondylolisthesis in the affected segment were included. Patients where additional dorsal instrumentation using pedicle screws and rods was performed, were also included. Patients who underwent multisegmental ALIF surgery and patients where additionally another interbody fusion procedure like transforaminal lumbar interbody fusion (TLIF) or posterior lumbar interbody fusion (PLIF) was performed were excluded. Altogether, 131 patients underwent ALIF surgery between 1.1.2013 and 10.12.2019. Thirty-five patients had to be excluded. In eight patients, a multisegmental ALIF was carried out. In nine patients, a combined procedure using ALIF and PLIF/TLIF was performed, and in one case, an intraoperative switch from ALIF to TLIF was performed. In 17 patients, there was no spondylolisthesis. Therefore, a total of 96 patients were included.

The assessed parameters were gender, age, BMI, type of procedure (standalone or additional dorsal instrumentation), diagnosis, type of interbody device used, type of instrumentation used, type of interbody graft used, duration of surgery, and complications. Vascular injury was defined as any injury of the iliac vessels. Regarding clinical outcomes, preoperative and postoperative (6 months after operation) Visual Analogue Scale (VAS; 0-100, 100 = maximum pain, 0 = no pain) for pain and Oswestry Disability Index (ODI; 0-100%, 100 = maximum disability, 0 = no disability) were assessed.

**Figure 1:**
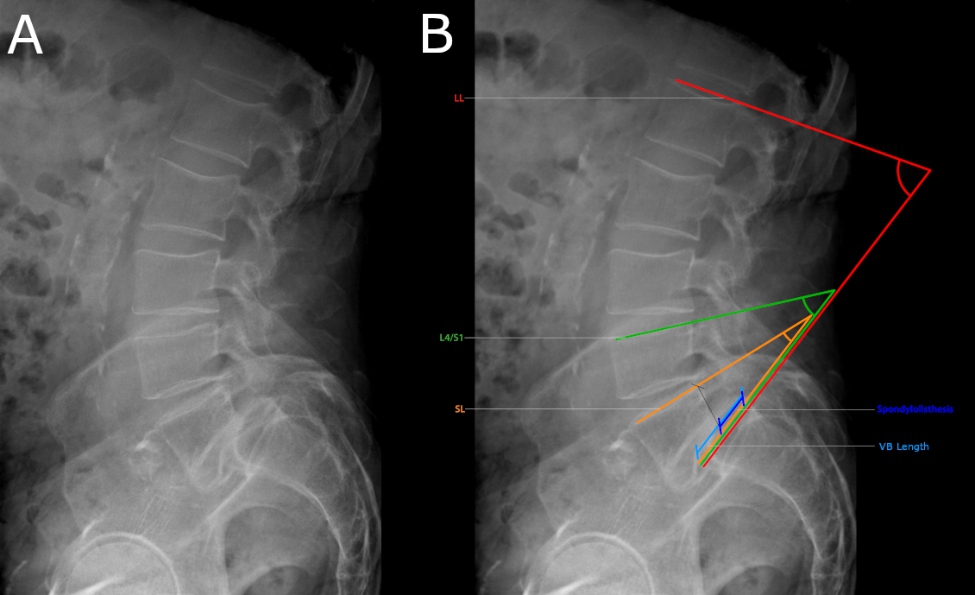
**(A)** Standing x-ray of the lumbar spine of a patient with spondylolisthesis at L5/S1. **(B)** Overview of the assessed radiological parameters. Measurements in this patient were: Lumbar lordosis (LL) = 72.1°; Segmental lordosis (SL) = 20.8°; L4/S1 lordosis (L4/S1) = 40.3°; Spondylolisthesis = 17.5 mm; Vertebral body length (VB Length) = 41.3 mm.

All radiographic measurements were taken from a lateral radiograph of the lumbar spine in a standing position (Fig. 1.A). All parameters were measured on preoperative and postoperative (6 months postoperative) x-rays. Assessed radiographic parameters include lumbar lordosis (LL), segmental lordosis (SL), L4/S1 lordosis, spondylolisthesis in mm, as well as the length of the upper endplate of the caudal vertebral body (see Fig. 1.B). LL was measured as the angle between the upper endplate of L1 and the upper endplate of S1; SL was measured as the angle between the upper endplates of the two fused vertebrae; L4/S1 lordosis was measured as the angle between the upper endplates of the L4 and S1. Spondylolisthesis was measured as the distance on the upper endplate of the lower vertebral body, measured from the dorsal end of the upper endplate of the lower vertebral body to the point where an imaginary extension of the dorsal edge of the upper vertebral body intersects with the endplate of the lower vertebral body. Length of the vertebral body is the length in mm of the upper endplate of the lower affected vertebral body. The relative subluxation of the upper vertebra against the caudal vertebra was calculated using the spondylolisthesis in mm and the vertebral body length of the caudal vertebra. The degree of subluxation was classified according to the Meyerding classification system [6].

### Surgical technique

#### ALIF

The patient is positioned supine under general anesthesia. First, a median, subumbilical skin incision or a Pfannenstiel incision was performed. Then, sharp dissection through the subcutaneous fat was performed. After incision of the anterior rectus sheath and dissection of the rectus abdominis muscle, the approach was continued using a retroperitoneal approach, usually on the left side, with dissection towards the promontory. After identification of the iliac arteries and veins, the vessels were mobilized, and the L5 and S1 vertebrae, along with the corresponding intervertebral disc, were visualized. These steps were performed by an access surgeon. A self-retaining retractor system was then fixed into the bone with pins. The correct height of the intervertebral disc L5/S1 was then verified under fluoroscopy. Then the disc space is revised, and intervertebral cage probes are inserted. After identifying a suitable cage size, another radiographic control was performed to verify the appropriate size and the depth of insertion. The final cage was then implanted and fixed with screws into the vertebral bodies of L5 and S1. A final anteroposterior and lateral fluoroscopy control was performed. Afterward, closure was performed using sutures for the fascia and subcutaneous tissue and staples for the skin.

#### Dorsal instrumentation

Additional dorsal instrumentation was performed after the anterior fusion procedure. Sterile drapes from the anterior procedure were removed after wound closure, and the patient position was switched then from supine to prone. An intraoperative x-ray system for 2D fluoroscopy and 3D imaging (O-arm™, Medtronic, Dublin, Ireland) was then set up. Using the fluoroscopy function of the O-arm™, the correct level was identified and, after skin disinfection and sterile draping, a median incision was performed, and the patient reference was then fixed to a spinous process. Intraoperative images were obtained and transferred to the navigation unit. The desired screw trajectories were then planned on the navigation unit. The skin incision was planned with the navigated pedicle access kit. The skin incision was then performed, and under navigational control, the PAK needle was inserted through the pedicle into the vertebral body. A K-wire was then inserted, and the PAK needle removed. After performing this for each screw position, another intraoperative scan was performed to verify the correct positioning of all K-wires. The screws were then inserted via the wires and brought into their final positions under fluoroscopic control. A final anteroposterior and lateral radiograph was taken. The navigation reference was then removed from the spinous process, followed by wound closure.

### Statistical analysis

Nominal variables are described with absolute and relative frequencies. Median and IQR (interquartile range) are reported for ordinal and non-normal metric variables and mean and standard deviation for normal metric variables. The normality of metric variables is tested with the Shapiro-Wilk-test. For variables measured at different time points, median and IQR are reported for the preoperative, the postoperative values, and their differences.

Differences in metric variables between standalone and dorsal instrumentation groups are tested with Mann-Whitney-U-tests. Spearman’s correlation coefficient (ρ) is used to describe the association between two non-normal metric variables, e.g., blood loss and operation time. Differences in metric variables such as VAS and ODI scores or lumbar and segmental lordosis are tested with the Wilcoxon-signed-rank-test.

Association between nominal variables measured preoperatively and postoperatively such as Meyerding grade is tested with a generalized McNemar’s test. Scores of VAS, ODI, LL, SL, and spondylolisthesis pre- and postoperatively are visualized by boxplots with lines showing changes of these measurements per individual. The level of significance was set to 0.05. The statistical software package R (version 4.1.0) was used for the statistical analysis [7].

## Results

Altogether, 96 patients – 55 female (57.29%), 41 male (42.71%) – were included. Baseline patient characteristics are shown in Table 1. The mean age was 51.98 years (SD: 11.44); the mean BMI was 27.36 (SD: 4.57). Main diagnoses were isthmic spondylolisthesis (n = 71, 73.96%), osteochondrosis (n = 14, 14.58%), degenerative spondylolisthesis (n = 4, 4.17%), post-laminectomy syndrome (n = 4, 4.17%), and adjacent segment degeneration (n = 3, 3.12%). In 84 cases (87.50%), additional dorsal instrumentation was performed, while in 12 cases (12.50%) standalone ALIF procedure was conducted. All procedures were carried out in the last lumbar segment – 93 in the L5/S1 segment, while three patients (3.12%) had a transitional vertebra with a sixth lumbar vertebra. For these patients, the ALIF procedure was carried out between L5 and L6. Operation time was assessed separately for the ventral fusion, for dorsal instrumentation, and total operation time. The median operation time for anterior interbody fusion was 83.00 min (IQR: 33.75); for dorsal instrumentation, it was 70.00 min (IQR: 35.00). The median total operation time for patients in which both anterior and dorsal surgery were performed was 142.00 min (IQR: 60.75).

**Table 1:** Baseline patient characteristics with n as absolute frequencies and % as relative frequencies. SD represents the standard deviation

Variable	n	%
Female	55	57.29
Male	41	42.71
Diagnosis		
• Isthmic spondylolisthesis	71	73.96
• Degenerative spondylolisthesis	4	4.17
• Osteochondrosis	14	14.58
• Adjacent segment degeneration	3	3.12
• Post-laminectomy syndrome	4	4.17
**Variable**	**Mean**	**SD**
Age	51.98	11.44
BMI	27.36	4.57

### Clinical outcome

**Figure 2:**
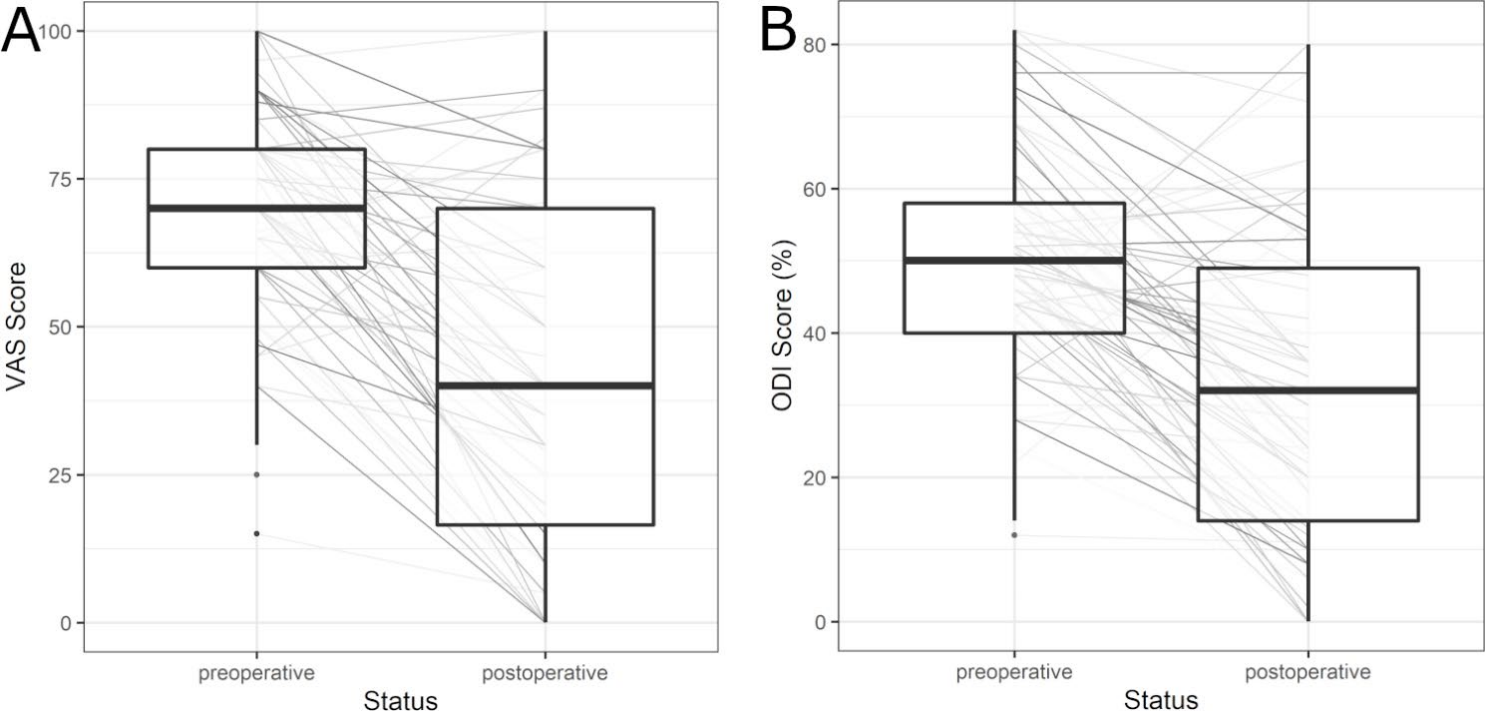
Development of the clinical parameters per individual (grey shaded lines) and grouped in the form of boxplots measured preoperatively and 6 months postoperatively. **A.** Development of Visual Analogue Scale (VAS). Median preoperative VAS = 70.00, median postoperative VAS = 40.00 (p < 0.001). **B.** Development of Oswestry Disability Index (ODI). Median preoperative ODI = 50.00%, median postoperative ODI = 32.00% (p < 0.001).

The median VAS score for preoperative pain was 70.00 (IQR: 20.00), while the median postoperative VAS score was 40.00 (IQR: 53.50) (median VAS reduction of 30.00, IQR: 40.00). The Wilcoxon signed-rank test shows a significant difference in VAS score pre- to postoperative (p < 0.001). The median preoperative ODI score was 50.00% (IQR: 18.00), and the median postoperative ODI score was 32.00% (IQR: 35.00), with a median reduction of 22.00% (IQR: 28.25). Again, the difference was significant (p-value < 0.001). Figure 2 shows the development of VAS (Fig. 2.A) and ODI (Fig. 2.B) per individual (grey shaded lines) and grouped in the form of boxplots measured preoperatively and postoperatively.

Mean blood loss was 384.38ml (median 150.00ml, IQR: 300.00ml). Blood loss was categorized into five groups. 31 (32.29%) patients had a blood loss lower than 50ml, 34 (35.42%) between 50 and below 250ml, 16 (16.67%) between 250 and below 500ml, 7 (7.29%) between 500 and less than 1000ml, and 8 (8.33%) of at least 1000ml.

Complications occurred in 18 patients. Absolute and relative numbers of complications are shown in Table 2. The most frequent complication was venous bleeding (n = 10, 10.42%), followed by wound healing disorders (n = 5, 5.21%). Arterial bleeding, implant loosening, deep vein thrombosis, retroperitoneal hematoma, and dura leak occurred once (1.04%). In two patients, both venous bleeding and wound healing disorder occurred. Revision surgery was performed due to postoperative complications in six patients (6.25%).

The occurrence of complications also led to a higher operation time, with median total operation times of 139.50 min (IQR: 60.25) in the group without complications and 204.00 min (IQR: 112.00) in the group with complications. The difference was significant (p = 0.0001). Higher blood loss was also significantly associated with a longer operation time (ρ = 0.37, p = 0.0002).

**Table 2:** Absolute number of complications and percentage of patients with a corresponding complication

Complication	Absolute number of complications	Percentage of patients (n = 96) with complication
Venous bleeding	10	10.42
Arterial bleeding	1	1.04
Wound healing disorder	5	5.21
Implant loosening	1	1.04
Deep vein thrombosis	1	1.04
Retroperitoneal hematoma	1	1.04
Dura leak	1	1.04

### Radiological outcome

**Figure 3:**
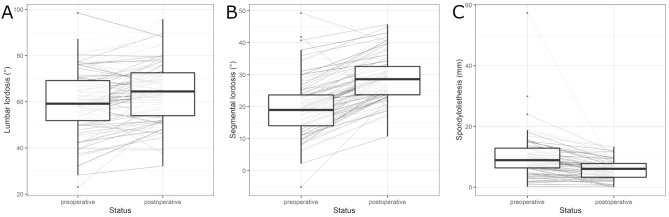
Development of radiological parameters per individual (grey shaded lines) and grouped in the form of boxplots measured preoperatively and 6 months postoperatively. **A.** Development of lumbar lordosis (LL). Median preoperative LL = 59.15°, median postoperative LL = 64.45° (p < 0.001). **B.** Development of segmental lordosis (SL). Median preoperative SL = 18.95°, median postoperative SL = 28.55° (p < 0.001). **C.** Development of spondylolisthesis. Median preoperative spondylolisthesis = 8.90 mm, median postoperative spondylolisthesis 6.05 mm (p < 0.001).

**Table 3:** gives an overview of the assessed radiological parameters. Median preoperative LL was 59.15° (IQR: 17.35°). Postoperatively, LL increased by a median of 4.20° (IQR: 10.25°) to 64.45° (IQR: 18.62°). Median preoperative SL was 18.95° (IQR: 9.65°) and increased by 9.50° (IQR: 5.47°) to 28.55° (IQR: 8.87°) postoperatively. The distribution of LL and SL is provided in addition to individual evolvements of the radiological parameters measured pre- and postoperatively in Fig. 3.A (LL) and 3.B (SL). Median preoperative L4/S1 lordosis was 37.90° (IQR: 11.93°) and increased by 6.00° (IQR: 6.40°) to 44.00° (IQR: 10.93°) postoperatively. Wilcoxon signed-rank tests yielded p-values < 0.001 for the differences between pre- and postoperative LL, SL, and L4/S1 lordosis

Variable	Median (IQR)
LL pre (°)	59.15 (17.35)
LL post (°)	64.45 (18.62)
LL diff (°)	4.20 (10.25)
SL pre (°)	18.95 (9.65)
SL post (°)	28.55 (8.87)
SL diff (°)	9.50 (5.48)
L4/S1 pre (°)	37.9 (11.93)
L4/S1 post (°)	44.00 (10.93)
L4/S1 diff (°)	6.00 (6.40)
VB pre (°)	40.05 (7.92)
VB post (°)	41.65 (6.95)
Spondylolisthesis pre (mm)	8.90 (6.50)
Spondylolisthesis post (mm)	6.05 (4.55)
Spondylolisthesis diff (mm)	-3.00 (4.08)
Spondylolisthesis pre (%)	21.63 (17.10)
Spondylolisthesis post (%)	13.71 (11.61)
Spondylolisthesis diff (%)	-7.47 (10.87)

Table 3. Preoperative, postoperative, and differences in pre- to postoperative measures are provided for lumbar lordosis (LL), segmental lordosis (SL), L4/S1 lordosis (L4/S1), and spondylolisthesis measured in mm as well as in %, respectively. For these variables, median and IQR (interquartile range) are reported.

Median vertebral body length on the preoperative lateral lumbar spine x-ray was 40.05 mm (IQR: 7.92), compared to 41.65 (IQR: 6.95) postoperatively. Median preoperative spondylolisthesis was 8.90 mm (IQR: 6.50) which was reduced to 6.05 (IQR: 4.55 mm) postoperatively (median absolute reduction of 3 mm (IQR: 4.08), median relative reduction of 33.71%). Details on this development of spondylolisthesis are provided in the form of boxplots and individual evolvements in Fig. 3.C. Relative slippage of the upper vertebra against the lower vertebra was 21.63% (IQR: 17.10) preoperatively and 13.71% (IQR: 11.61) postoperatively. Preoperatively, 58 patients were categorized as Meyerding grade I, 36 as Meyerding grade II, one as Meyerding grade III, and one as Meyerding grade IV. Postoperatively, there were 83 patients with Meyerding grade I and 13 patients with Meyerding grade II. No patients were classified as Meyerding grade III or IV postoperatively (Table 4). A generalized McNemar’s test was used to test whether the distribution of the Meyerding grades before and after the operations are different. The corresponding p-value is smaller than 0.001 and shows that a significant change in the distribution was observed.

**Table 4:** Absolute values of Meyerding grade preoperative (“pre”) and postoperative (“post”)

	Meyerding I (post)	Meyerding II (post)	Meyerding III (post)	Meyerding IV (post)	Sum
Meyerding I (pre)	58	0	0	0	58
Meyerding II (pre)	24	12	0	0	36
Meyerding III (pre)	1	0	0	0	1
Meyerding IV (pre)	0	1	0	0	1
Sum	83	13	0	0	96

### Comparison of patients with standalone ALIF vs. additional dorsal instrumentation

Patients who underwent ALIF standalone are compared to patients who also received additional dorsal instrumentation. Neither the change of ODI (median (IQR); dorsal instrumentation, n = 57: -24.00 (26.00); standalone, n = 7: -8.00 (24.50); p = 0.3953) nor of VAS significantly differed (median (IQR); dorsal instrumentation, n = 53: -35.00 (42.00), standalone, n = 6: -15.00 (26.50); p = 0.2531) between the groups. Hence, the tendency of better results of the dorsal instrumentation group compared to the standalone group is not significantly different from zero. No significant association between complications and the performance of standalone procedures is observed (p-= 0.4536).

The comparison of the standalone group (n = 12) and the dorsal instrumentation group (n = 84) revealed that the pre- to postoperative differences in LL (p = 0.6941), SL (p = 0.9602), and L4/S1 lordosis (p = 0.7147) did not differ significantly between the groups. The observed difference of the correction of spondylolisthesis between the standalone group and the dorsal instrumentation group is not significant (p = 0.0829).

## Discussion

We present the results of 96 patients who underwent ALIF for degenerative spine pathologies where spondylolisthesis was present. In most cases, the procedure was combined with additional dorsal instrumentation using pedicle screws and rods.

Most patients (n = 94, 97.92%) were not rated as having high-grade spondylolisthesis (Meyerding III or IV). This finding is in accordance with the results of other studies, as all our patients were adults with degenerative diseases of the spine, which are typically associated with lower grades of spondylolisthesis. Viglione et al. (2017) state that adult low-grade spondylolisthesis and adolescent high-grade spondylolisthesis should be clearly distinguished, suggesting different pathological entities in these age groups [8]. High-grade spondylolisthesis typically develops in adolescents with isthmic spondylolisthesis when the posterior arch is not completely ossified and the intervertebral disc is very elastic. Low-grade spondylolisthesis is more common in older patients when the intervertebral disc is less elastic due to degenerative changes [8–10].

In our sample, median spondylolisthesis was significantly reduced postoperatively, by 3.00 mm (IQR: 4.08), along with a reduction of the relative subluxation from 21.63% (IQR: 17.10) to 13.71% (IQR: 11.61). This is a 33.71% reduction from the preoperative value. Our findings are similar to the reductions reported in the literature. In 2020, Kalani et al. reported a mean reduction of spondylolisthesis by 58.7% in combined procedures with anterior fusion and dorsal instrumentation [11]. Rao et al. (2015) detected a reduction from 14.8% in preoperative spondylolisthesis, measured as the relative subluxation, to 9.4% at the latest follow-up [2]. Tu et al. (2021) and Riouallon et al. (2013) report a mean spondylolisthesis reduction of 30% [3, 4]. In a meta-analysis of 2019, Cho et al. [12] compared anterior and posterior approaches for spondylolisthesis treatment. Only three out of eight studies reported the degree of spondylolisthesis [13–15] after anterior fusion procedures. In these studies, mean preoperative slippage was 19.8% (Meyerding grade I), and this was postoperatively reduced to a mean slippage of 7.8% (12% points difference). With a mean reduction of 8%, the achieved slippage reduction was similar in our sample. Comparing our sample with posterior-only techniques, Moreau et al. (2016) report a decrease of spondylolisthesis by 50% using a posterior-only fusion technique for L5/S1 spondylolisthesis cases and therefore a similar reduction rate of spondylolisthesis [16].

Most studies investigating the reduction of spondylolisthesis after ALIF have relatively low sample size numbers, with case numbers between 5 and 65 participants [3–5, 11, 13, 14, 17] and most studies having fewer than 30 participants [4, 5, 11, 14, 17]. With 96 cases, we present, to our knowledge, one of the most extensive single-center studies investigating this topic.

Additional dorsal instrumentation did not significantly change the lordosis values’ postoperative outcome or the amount of spondylolisthesis reduction. As in most cases of spondylolisthesis, dorsal instrumentation is recommended for reasons of stability and good results concerning bony fusion. Studies comparing standalone procedures with dorsal instrumentation are scarce, and to our knowledge, we present one of the first studies to show that the anterior procedure is the main contributor to the improvement of spondylolisthesis and lordosis.

Knowledge and research on the sagittal alignment of the spine have multiplied over the last few years. Sagittal alignment and LL are of significant importance when treating patients with spondylolisthesis [12], as sagittal malalignment and loss of LL can lead to chronic lower back pain [18]. Cho et al. (2019) found in their meta-analysis that LL and SL were significantly higher after anterior approach with dorsal instrumentation procedures compared to posterior fusion techniques with dorsal instrumentation [12]. In our study, median lordosis increased significantly, with a median increase in LL of 4.20° (IQR: 10.25), in SL median of 9.05° (IQR: 5.48), and in L4/S1 lordosis median of 6.00° (IQR: 6.40). Again, these findings are similar to those reported in the literature. Moreau et al. (2016) found an LL increase of 7° [16] and Caprariu et al. (2021) an increase of 8° [16, 17]. Kalani et al. detected increases in mean SL, defined as L4–S1 lordosis, and overall (L1–S1) LL after ALIF with dorsal instrumentation of 23.6% and 16.6%, respectively [11].

The finding that L4/S1 and SL in the operating segment are postoperatively more increased than the total LL might indicate that the lumbar spine as a whole partly compensates for correction of the SL within the segment where fusion was performed. However, further research on this topic should be conducted, as specific physiological mechanisms are not described in the literature leading to these results.

A meta-analysis from 2016 comparing complication rates in ALIF and extreme lateral interbody fusion procedures found an overall complication rate of 26.47% for ALIF versus 16.61% for extreme lateral interbody fusion [19]. Most of the complications were neurological, like motor weakness, hypoesthesia, or thigh symptoms, although almost half (48.1%) of all neurological complications resolved within 42 days. Revision surgery was performed in 4.60% of all ALIF procedures, with the most frequent reason being pseudarthrosis, followed by hardware failure. The wound infection rate was 5.75% [19]. In our sample, the most frequent complication was an intraoperative injury to venous structures during the anterior approach. Rates of vascular injury vary between different studies. A recent review found a venous injury rate of 10.4%, similar to the rate we found in our study [20]. Another review reports a wide range of vascular injury rates, from 0% up to 18.1%, with arterial injuries being less frequent than venous lacerations [21]. Mean blood loss was 384.38ml in our sample (median 150ml). This is similar to blood loss values reported in the literature. Tu et al. (2021), for example, found a mean blood loss of 300ml, and Teng et al. (2017) report a mean blood loss of 200-300ml, which, however, can increase drastically in the case of vascular injury [4, 22]. Blood loss associated with venous vessel injury ranged from 250ml to 10,000ml in our sample. The four patients with venous injuries had estimated blood losses of 1,000ml or higher. The patient with an arterial injury had a blood loss of 1,400ml. When considering the potentially fatal consequences of vascular injury, one of the major limitations of ALIF is evident. Care should be taken especially to rule out a relatively low level of the bifurcation of the abdominal aorta and the confluence of the inferior vena cava. Inamasu et al. investigated the level of the aortic bifurcation and found a low bifurcation level in 18% with the bifurcation at the height of the vertebral body L5. Considering the level of the confluence of the inferior vena cava, they found a rate of 1% of all cases where the confluence is located at L5/S1 or below [23]. Surgical access can be complicated when encountering the vascular structures and especially any vascular bifurcation directly ventral of the L5/S1 intervertebral disc. Before indicating ALIF surgery, individual vascular anatomy should be examined on appropriate radiological examinations like magnetic resonance imaging (MRI) or computer-assisted tomography (CT) to minimize the risk of vascular injury.

Our study was limited by several factors. First, the results may be biased due to the retrospective study design. Also, the sample size of the patients who underwent standalone ALIF was relatively small. We recommend conducting prospective, multi-center studies with large sample sizes to investigate sagittal alignment and spondylolisthesis after ALIF surgery.

## Conclusions

Good clinical results can be achieved with ALIF, and effective reduction of spondylolisthesis and restoration of LL is possible. We present one of the most extensive single-center studies investigating this topic. Additional dorsal instrumentation did not significantly affect postoperative lordosis or spondylolisthesis compared to the standalone group. As vascular injury is a frequent complication with potentially fatal consequences, individual vascular anatomy must be analyzed on MRI or CT before considering ALIF. Therefore, in patients where the fusion of the lower lumbar spine is indicated in the presence of spondylolisthesis and suitable anatomy, we recommend the performance of ALIF surgery combined with additional dorsal instrumentation.

## Data Availability

The datasets used and/or analysed during the current study are available from the corresponding author on reasonable request.
